# The Metabolic Transition Between Fasting and Feeding Alters Aging‐Associated Metabolites, Lowers BCAAs, and Stimulates FGF21 Production in Humans

**DOI:** 10.1111/acel.70270

**Published:** 2025-10-21

**Authors:** Maria Lastra Cagigas, Nancy T. Santiappillai, Serena Commissati, Giovanni Fiorito, Andrius Masedunskas, Gayathiri Rajakumar, Isabella de Ciutiis, Alan Goldhamer, Brian K. Kennedy, Andrew J. Hoy, Luigi Fontana

**Affiliations:** ^1^ Charles Perkins Centre, Faculty of Medicine and Health The University of Sydney Camperdown Australia; ^2^ Sydney Medical School Nepean, Faculty of Medicine and Health The University of Sydney Camperdown Australia; ^3^ School of Medical Sciences, Faculty of Medicine and Health The University of Sydney Camperdown Australia; ^4^ Department of Radiology Memorial Sloan Kettering Cancer Center New York New York USA; ^5^ Molecular Pharmacology Program Memorial Sloan Kettering Cancer Center New York New York USA; ^6^ Department of Geriatrics Ca' Foncello Treviso Hospital Treviso Italy; ^7^ Clinical Bioinformatics Unit IRCCS Istituto Giannina Gaslini Genoa Italy; ^8^ TrueNorth Health Center Santa Rosa California USA; ^9^ Healthy Longevity Translational Research Programme, Yong Loo Lin School of Medicine National University of Singapore Singapore Singapore; ^10^ Centre for Healthy Longevity National University Health System Singapore Singapore; ^11^ Department of Biochemistry and Physiology, Yong Loo Lin School of Medicine National University of Singapore Singapore Singapore; ^12^ Department of Endocrinology Royal Prince Alfred Hospital Sydney Australia

**Keywords:** BCAA, fasting, FGF21, ketogenesis, refeeding

## Abstract

Fasting‐based interventions are gaining momentum as strategies to modulate longevity. Conversely, the same metabolic adaptations that once ensured survival during starvation now contribute to the global obesity epidemic. While previous studies have characterized metabolic changes during fasting, few have examined the refeeding phase, and most lack an integrated analysis of key hormonal and metabolic regulators, including insulin, leptin, adiponectin, free T3, FGF21, and the plasma metabolome. To address this gap, we profiled 134 plasma metabolites using mass spectrometry, covering pathways involved in lipid, amino acid, and ketone metabolism, in a cohort of 20 adults (mean age 52.2 ± 11.8 years, 55% women, BMI 28.8 ± 6.4 kg/m^2^) undergoing medically supervised prolonged fasting (mean duration 9.8 ± 3.1 days), followed by plant‐based refeeding (5.3 ± 2.4 days). Fasting reduced metabolic rate, reflected by lower free T3 levels (*p* < 0.0001), and markedly reprogrammed the plasma metabolome, including shifts in seven aging‐associated metabolites (glucose, 3‐hydroxybutyric acid, glycine, glutamine, alanine, phenylalanine, and tyrosine). Notably, plasma branched‐chain amino acid (BCAA) levels remained stable during fasting, suggesting active tissue release to support energy homeostasis alongside ketogenesis. Upon refeeding, 81% of metabolite levels normalized, yet BCAAs declined sharply (valine −45%, leucine −52%, isoleucine −48%; all *p* < 0.001), consistent with insulin‐stimulated tissue uptake. Changes in BCAAs were inversely associated with a fivefold increase in FGF21 levels (243.2–1176 pg/mL, *p* = 0.0007), which occurred exclusively during refeeding, unlike in rodent models where FGF21 levels rise during fasting. Together, our findings identify refeeding as a critical window for modulating aging‐related metabolites and highlight the importance of post‐fast refeeding dynamics.

## Graphical abstract

In humans, prolonged fasting profoundly remodels the plasma metabolome while maintaining stable levels of BCAA and FGF21. Upon refeeding, plasma BCAAs (valine, isoleucine, and leucine) decline, reflecting tissue uptake and coinciding with a surge in FGF21, independent of insulin and glucose.

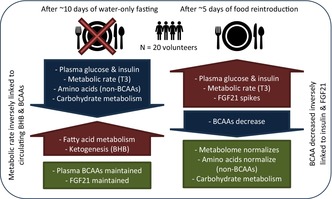

## 1 Introduction

Throughout evolutionary history, periods of famine have shaped human metabolism, driving the development of metabolic plasticity, the ability of cells to dynamically regulate nutrient utilization in response to changing energy availability (Commissati et al. 2025; Folmes et al. 2012). This adaptability is particularly evident during prolonged fasting, when the body transitions from glucose metabolism to fatty acid oxidation and ketogenesis to sustain energy homeostasis. While these metabolic adaptations were once vital for survival, they now contribute to the obesity epidemic, as mechanisms evolved to counteract energy scarcity paradoxically promote metabolic dysfunction in an environment of chronic caloric excess (Ampofo and Boateng 2020).

Emerging evidence identifies dysregulated branched‐chain amino acids (BCAAs) and fibroblast growth factor 21 (FGF21) as key biomarkers of early metabolic decline (Zhang et al. 2024). Elevated circulating BCAA levels are strongly associated with insulin resistance and an increased risk of type 2 diabetes, implicating their role in impaired glucose metabolism (Zhang et al. 2024). In contrast, FGF21 has been recognized as a critical regulator of energy balance, enhancing glucose uptake, improving insulin sensitivity, and promoting lipid oxidation (Buergel et al. 2022). Despite their recognized importance, the interplay between BCAAs, FGF21, and other metabolic regulators during fasting and refeeding remains poorly understood, with limited studies integrating fasting‐induced hormonal signals and high‐dimensional metabolomics in humans.

To address this gap, we investigated the effects of a medically supervised fasting and refeeding intervention on the circulating metabolome of 20 adults across a range of BMI categories in both sexes. Given that the circulating metabolome serves as a robust predictor of metabolic dysfunction and mortality (Zhang et al. 2024; Buergel et al. 2022; Talmor‐Barkan et al. 2022), we examined how key endocrine regulators (insulin, leptin, adiponectin, triiodothyronine (T3), and cortisol) interact with glucose, amino acids, fatty acids, and ketone bodies to shape systemic metabolic responses during fasting and refeeding. Furthermore, we explore the interrelationship between BCAAs and FGF21 as critical biomarkers of metabolic health and aging (Choi et al. 2024; Luo et al. 2017), providing new insights into metabolic adaptations that may inform therapeutic strategies for obesity‐related disorders.

## 2 Results

We studied 20 adult volunteers (age 52.2 ± 11.8 years, 55% women, BMI 28.8 ± 6.4 kg/m^2^) at three time points: (i) baseline, (ii) after an average of 9.8 days (9.8 ± 3.1 days) of prolonged water‐only fasting, and (iii) following 5.3 ± 2.4 days of gradual refeeding, as previously described. (Commissati et al. 2025) The cohort encompassed a spectrum of cardiometabolic profiles (Table 1): normoweight and normoglycaemic (*n* = 4; BMI < 25 kg/m^2^ and glucose < 100 mg/dL), overweight and/or hyperglycaemic (*n* = 10; BMI 25–30 kg/m^2^ or glucose > 100 mg/dL), and obese (*n* = 6; BMI > 30 kg/m^2^). Healthy participants were younger on average (41.5 years) and had smaller waist circumference (80.5 cm), and lower fasting glucose (81.5 mg/dL), triglycerides (68.8 mg/dL), and blood pressure (115/70 mmHg). By contrast, individuals with obesity exhibited higher fasting insulin (14.7 μU/mL), triglycerides (128.3 mg/dL), and blood pressure (128/73 mmHg). Endocrine profiling further indicated low FGF21 and leptin concentrations in normoweight and overweight groups, while obesity was associated with markedly elevated FGF21 (415.2 pg/mL) and leptin, together with reduced adiponectin (4475.7 ng/mL) (Table 1).

**TABLE 1:** Baseline cardiometabolic and endocrine status across volunteers.

	Full cohort (*n* = 20)		Healthy (*n* = 4)		Overweight (*n* = 10)		Obesity (*n* = 6)	
			(BMI < 25 kg/m^2^ and glucose < 100 mg/dL)		(BMI 25–30 kg/m^2^ or glucose > 100 mg/dL)		(BMI > 30 kg/m^2^)	
	Mean	SD	Mean	SD	Mean	SD	Mean	SD
Sex, female (%)	11 (55%)	—	2 (50%)	—	4 (40%)	—	5 (83%)	—
Age (years)	52.2	11.8	41.5	11.0	52.6	12.4	58.7	3.1
Height (cm)	173.4	10.8	169.3	11.9	175.5	10.9	175.6	8.9
Weight (kg)	86.6	20.6	65.7	6.0	82.2	13.2	107.9	17.9
BMI (kg/m^2^)	28.8	6.4	23.0	1.1	26.5	1.6	36.4	6.5
Glucose (mg/dL)	85.7	10.4	81.5	7.1	84.0	11.4	91.2	8.0
Insulin (μU/mL)	7.6	6.5	5.4	2.3	4.26	2.39	14.7	7.5
Triglycerides	102.8	46.2	68.8	20.6	101.1	44.3	128.3	46.2
HDL cholesterol	56.8	19.2	62.3	21.5	57.7	19.4	51.5	15.6
LDL cholesterol	108.4	37.4	99.0	24.3	108.7	46.6	114.2	23.4
Total cholesterol	192.0	33.5	175.3	33.1	199.2	33.7	191.2	29.2
SBP	123.9	10.2	115.3	7.8	125.0	10.8	127.7	6.8
DBP	72.1	8.7	70.0	9.7	72.4	8.7	73.1	7.9
Waist (cm)	96.4	12.7	80.5	4.1	95.0	8.2	109.4	8.6
Free T3 (pg/mL)	3.3	0.3	3.6	0.3	3.2	0.3	3.2	0.1
FGF‐21 (mg/mL)	243.2	193.0	95.5	75.4	199.0	144.8	415.2	195.0
Leptin (μg/L)	52.7	59.9	22.0	18.4	22.6	14.2	123.4	65.2
Adiponectin (ng/mL)	5643.4	3199.3	6708.0	3627.8	5918.2	3349.0	4475.7	2099.1

Following an overnight fast, plasma levels of 419 untargeted metabolites were measured using state‐of‐the‐art ALEX‐CIS GC‐TOF mass spectrometry, identifying 134 known metabolites and 285 unknowns (Methods). Fasting adherence was high, with all participants exhibiting hallmark starvation physiology, including significant weight loss (6.64 ± 1.85 kg, representing 7.6% of body weight, *p* < 0.0001), a marked rise in plasma β‐hydroxybutyrate (BHB) levels (from 0.6 ± 0.9 to 5 ± 1 mmol/L, *p* < 0.0001), and a reduction in glucose concentrations (from 85.7 ± 10.4 to 70.3 ± 10.5 mg/dL, *p* = 0.0002), as previously reported (Commissati et al. 2025). The fasting‐induced ketosis was reversed upon refeeding. Most participants tolerated the protocol well; however, fasting was discontinued at the participant's request or due to medical necessity. Six participants transitioned to a vegetable broth or juice fast (< 300 kcal/day) due to side effects such as nausea, fatigue, and headache, as previously reported (Commissati et al. 2025). For these individuals, blood samples collected prior to the transition were used to represent the fasting time point. One participant (ID05) consumed juice prior to blood collection and was therefore excluded from the metabolomics analysis.

### 2.1 Fasting Promotes Fatty Acid Oxidation, Ketone Body Metabolism, and Amino Acid Degradation

**FIGURE 1:**
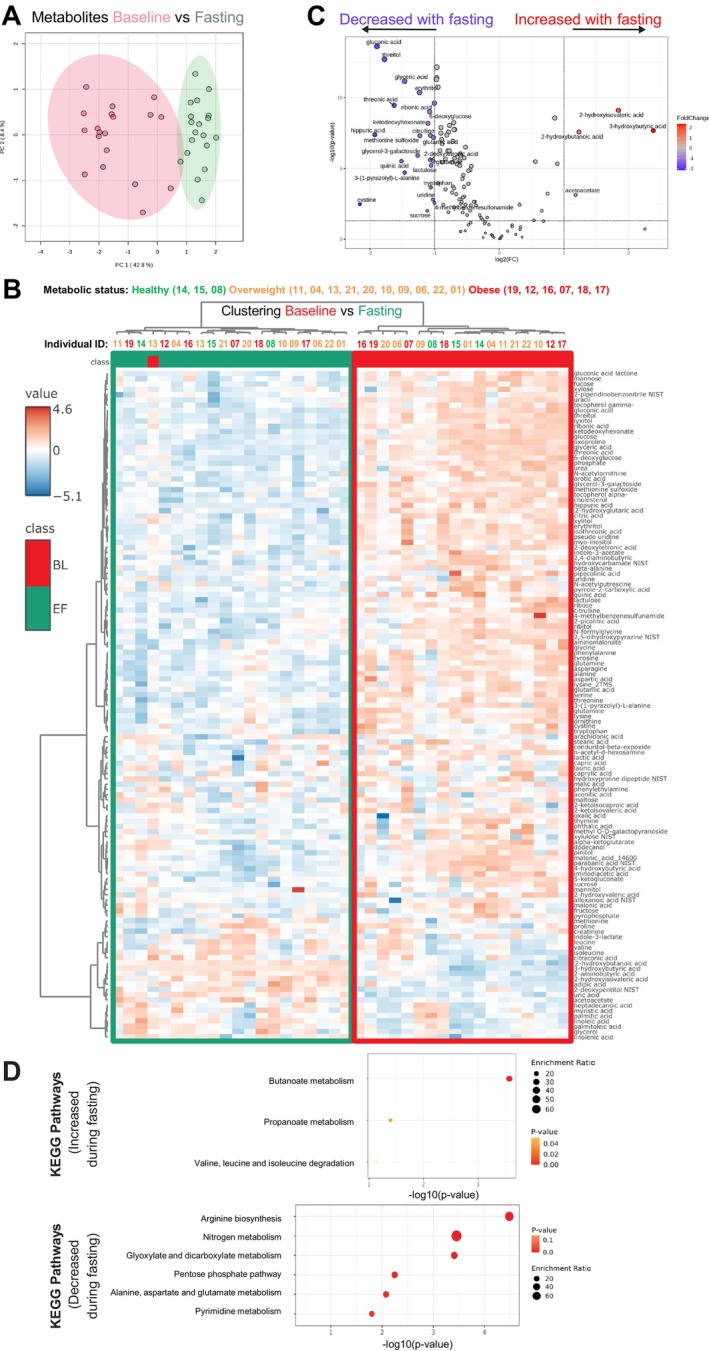
Fasting promotes fatty acid oxidation, ketone body metabolism, and amino acid degradation. (A) PCA for the levels of 134 identified metabolites in blood at Baseline (BL) and End of Fasting (EF). Overlap in colored areas denotes similar metabolite levels, whereas separation of denotes differences in metabolite levels. (B) Heatmap clustering of the same metabolites, showing distinctive clustering between BL (red) and EF (green). Stratification by cardiometabolic status (healthy—green, overweight/hyperglycaemic—orange, or obese—red) did not show clustering by metabolic status in the response to fasting. (C) Volcano plots of the same 134 metabolites. Significantly changed metabolites (FDR < 0.05, log2(FC) > |1|) are shown in blue (decreased) and red (increased). (D) KEGG pathway enrichment analysis of differentially abundant metabolites. Pathways significantly altered (*p* < 0.05) are shown. *N* = 19 volunteers (one noncompliant excluded) for all graphs.

**FIGURE 2:**
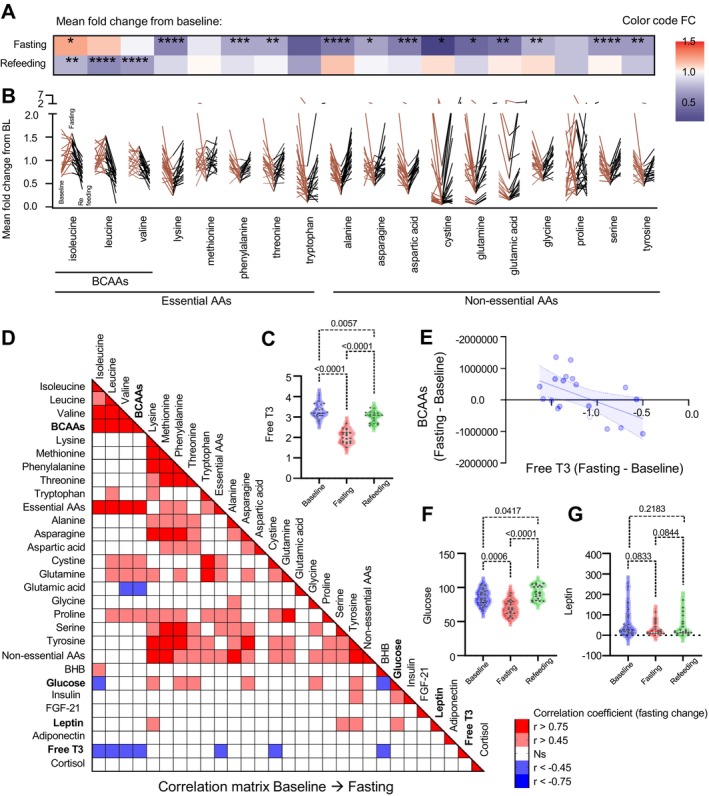
Lower metabolic rate correlates with increased BCAA release and deeper ketosis. (A) Heatmap of normalized mean fold change in plasma amino acid levels relative to baseline. Colors represent increased (red) or decreased (blue) amino acid levels, and asterisks denote *p* values, * < 0.05, ** < 0.01, *** < 0.001, **** < 0.0001 calculated using paired two‐way ANOVA with correction for multiple comparisons. (B) Same results as in (a), showing the fold change in plasma amino acid levels for each volunteer from baseline (BL) to the End of Fasting (EF/BL) and End of Refeeding (ER/BL). Each line represents a volunteer. *N* = 18 amino acids (arginine and histidine not detected). (C) Circulating levels of free T3 at three timepoints. Statistical significance was calculated using paired two‐way ANOVA and Turkey's correction for multiple comparisons. *p* values are shown. No outliers were present. (D) Correlation matrix showing Pearson's correlation coefficients (red square = significant positive correlation, blue square = significant negative correlation, *p* value < 0.05) between multiple delta variables (End of Fasting [EF]—Baseline [BL]). Matrix was corrected for outliers. (E) Linear regression between delta free T3 and delta BCAAs. Individual dots represent participants. Shaded areas represent 95% CI for the best‐fit line. (F, G) Circulating levels of glucose and leptin at three timepoints. Statistical significance was calculated using paired two‐way ANOVA and Turkey's correction for multiple comparisons. *p* values are shown. Eight datapoints were outliers for leptin; graph without the outliers is shown in Figure S6.

To examine the impact of prolonged fasting on the circulating metabolome, we performed principal component analysis (PCA) of plasma metabolite levels, revealing a clear separation between baseline and fasting samples (Figure 1A) with no visual outliers, indicating a relatively homogeneous response independent of metabolic status. Similarly, unsupervised hierarchical clustering using Ward's method demonstrated distinct separation, with all fasting samples and one baseline outlier clustering together (Figure 1B). Stratification by cardiometabolic status (healthy, overweight/hyperglycaemic, or obesity; Table 1 and Figure 2B) did not reveal distinct metabolomic clustering. Participants from all three groups clustered together both at baseline (e.g., volunteers 11, 19, and 14) and after fasting (e.g., volunteers 09, 08, and 18). This suggests that, while cardiometabolic status may influence the fasting response, the effect of fasting itself was more pronounced than inter‐individual differences in metabolic health.

Overall, fasting significantly altered 23% (31 of 134) of the identified metabolites (log2(FC) > 1, FDR < 5%), with four increasing more than twofold and 27 decreasing by over 50% (Figure 1C). Among the metabolites that increased, two were ketone bodies synthesized from fatty acid oxidation in hepatocyte mitochondria (3‐hydroxybutyric acid and acetoacetate), while the other two were intermediates of amino acid metabolism (2‐hydroxyisovaleric acid and 2‐hydroxybutyric acid). In contrast, 27 metabolites, primarily sugars (e.g., gluconic acid and erythritol), amino acids (e.g., citrulline and glutamic acid), and organic acids (e.g., hippuric acid and quinic acid), significantly declined (log2(FC) < −1, FDR < 5%). These findings suggest a fasting‐induced metabolic shift favoring the production of ketone bodies. Notably, this response differs from shorter fasting durations (1.4–2.4 days), where most metabolites tend to increase. (Teruya et al. 2019) Although previous studies have reported sex‐specific fasting responses, with women relying more on fat oxidation and men on gluconeogenesis, our study found no significant sex differences in overall metabolite levels (Figure S1). However, the sample size (11 women and 9 men) was underpowered to robustly assess sex effects. Regardless, clustering analysis suggested a potentially more homogeneous metabolic response among women, as their fasted metabolite profiles clustered more closely (Figure S1B).

To investigate pathway alterations, we used Kyoto Encyclopedia of Genes and Genomes pathway enrichment maps (KEGG, Figure 1D), literature‐based associations (CompBio, Figure S2), and curated experimental data (Ingenuity Pathway Analysis, Figure S2). Fasting significantly upregulated (pathway *p*‐value < 0.05) ketone body metabolism (ketogenesis and ketolysis), short‐chain fatty acid (SCFA) metabolism (butanoate and propanoate), and branched‐chain amino acid (BCAA) degradation (valine, leucine, and isoleucine) pathways. These findings align with a prior study showing decreased BCAA levels following a 60‐h fast (Schauder et al. 1985). Conversely, fasting significantly downregulated (pathway *p*‐value < 0.05) amino acid synthesis (arginine) and metabolism (alanine, aspartate, and glutamate). Carbohydrate metabolism was also suppressed via the pentose phosphate pathway (PPP) (Figure 1D). This metabolic suppression correlated with the downregulation of the somatotrophic/growth hormone axis (Figure S2). In summary, consistent with previous findings (Pietzner et al. 2024; Schauder et al. 1985; Steinhauser et al. 2018; Buono and Longo 2018), the circulating metabolome following prolonged fasting reflected adaptations that promote ketone utilization, enhance fatty acid oxidation, and suppress carbohydrate metabolism and protein synthesis.

### 2.2 Lower Metabolic Rate Correlates With Increased BCAA Release and Deeper Ketosis

Pathway analyses revealed alterations in amino acid metabolism during fasting. Longitudinal profiling of plasma amino acids (*n* = 18; histidine and arginine were not detected) showed significant declines in the majority of essential and nonessential amino acids (12 amino acids, all adj *p* < 0.05), with uniform reductions observed across participants (Figure 2A,B), in line with previous research (Schauder et al. 1985; Felig et al. 1969; Sherwin 1978). In contrast, levels of methionine, proline, leucine, and valine remained unchanged (non‐significant *p* values), showing no significant differences after the prolonged fast. Isoleucine displayed an opposite and distinct pattern, with a positive fold change (adj *p* < 0.05) during fasting (Figure 2A). This finding aligns with prior preclinical studies, including a rat study that reported maintained or increased plasma BCAA levels after 3 days of starvation (Holecek et al. 2001), attributed to reduced BCAA catabolism in heart and skeletal muscles, as well as a net release of BCAA from the liver. Another study found a two‐ to three‐fold increase in the net release of leucine from forearm muscle in individuals fasting for 60 h (Fryburg et al. 1990), suggesting that both the liver and muscle contribute to BCAA release during prolonged fasting. Thus, our findings corroborate previous research on starvation (Felig et al. 1969; Sherwin 1978; Owen et al. 1969; Garber et al. 1974), confirming that plasma BCAA levels remain either stable or elevated even after 10 days of energy deprivation.

Notably, individual‐level changes in BCAAs were significantly correlated with alterations in free T3, a critical thyroid hormone involved in regulating basal metabolic rate (Sui et al. 2024). Fasting significantly lowered free T3 levels, from 3.292 to 2.051 pg/mL (adj *p* < 0.0001) (Figure 2C), and this reduction inversely correlated with plasma BCAA levels (Pearson *r* = −0.65, *p* = 0.0019) (Figure 2D,E and Figure S3). This suggests that prolonged fasting reduces metabolic rate in synchrony with BCAA release. Free T3 changes were also closely linked to BHB levels (Pearson *r* = −0.513, *p* = 0.021) (Figure 2D and Figure S3). Significant correlations are shown in Figure S3. By contrast, no significant correlations were observed between BCAA fluctuations and other major metabolic regulators, including glucose, insulin, FGF21, leptin, adiponectin, and cortisol (Figure 2D). Together, these findings reveal a novel interaction between thyroid hormones, BCAAs, and ketone metabolism, highlighting a coordinated regulation of whole‐body energy balance during fasting with potential implications for muscle preservation.

In addition to BCAAs, fluctuations in other amino acids also correlated with key metabolic regulators. Specifically, changes in plasma glucose and leptin levels, which decreased during fasting from 85.65 to 70.25 mg/dL (adj *p* = 0.0006) (Figure 2F) and 52.72 to 32.61 ng/mL (adj *p* = 0.0833) (Figure 2G), respectively, correlated with multiple amino acids (Figure 2D). The strongest correlations (all *p* < 0.05) were observed between changes in leptin and tyrosine (Pearson *r* = 0.60), serine (*r* = 0.54), and lysine (*r* = 0.47), and between changes in glucose and lysine (Pearson *r* = 0.57), threonine (*r* = 0.58), and asparagine (*r* = 0.55). These results are consistent with previous in vitro studies demonstrating that amino acid levels regulate leptin secretion from white adipocytes (Cammisotto et al. 2005), highlighting the potential for amino acid‐based precision nutrition to influence glucose and leptin levels in metabolic interventions.

### 2.3 Post‐Fast Refeeding Restores Metabolites and Increases Insulin and BCAA Uptake

**FIGURE 3:**
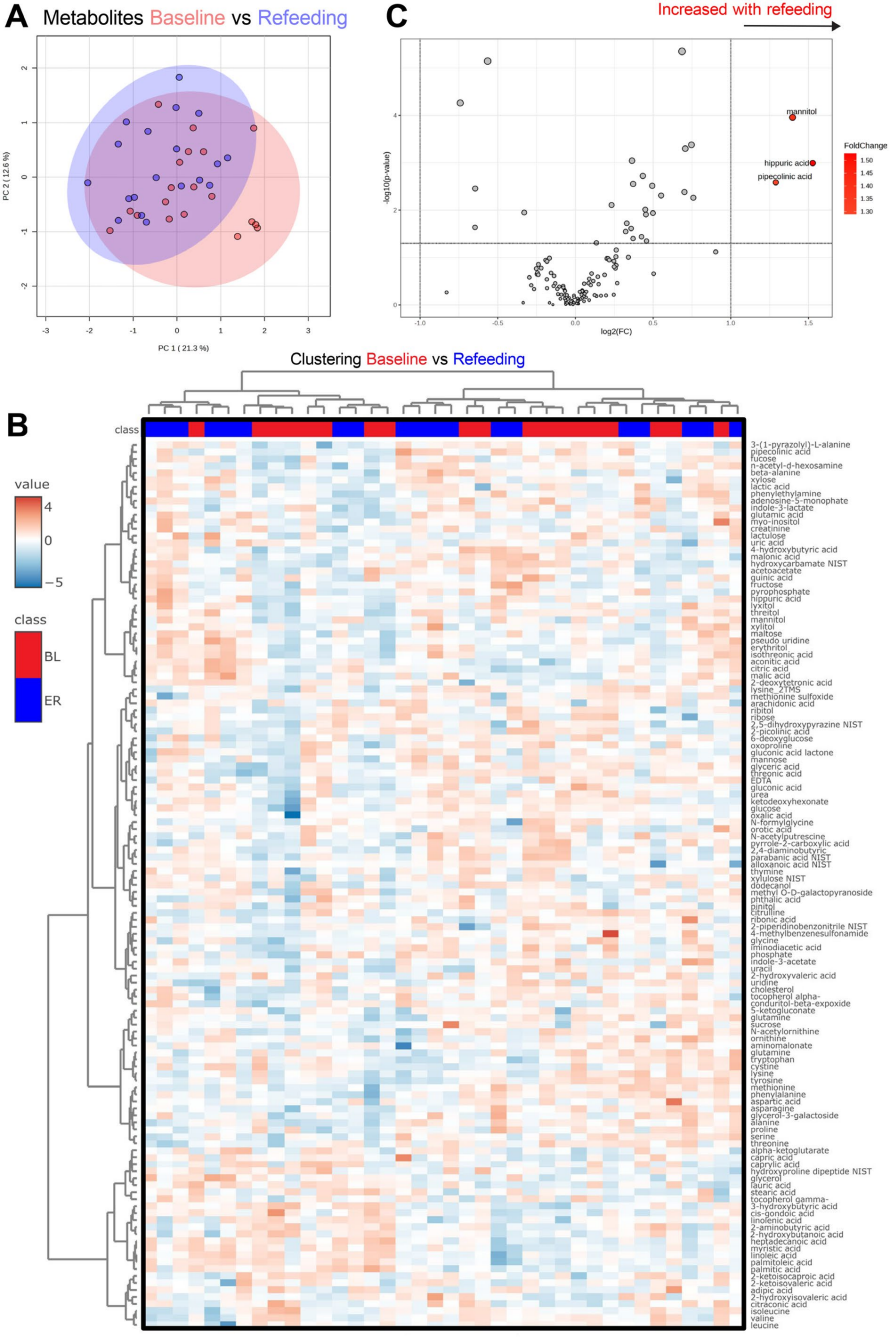
Post‐fast refeeding restores metabolites and facilitates insulin‐mediated BCAA uptake. (A) PCA for the levels of 134 identified metabolites in blood at Baseline (BL) and End of Refeeding (ER). Overlap in colored areas denotes similar metabolite levels, whereas separation of denotes differences in metabolite levels. (B) Heatmap clustering of the same 134 metabolites, showing a lack of distinctive clustering between BL and ER. (C) Volcano plots of the same 134 metabolites. Significantly changed metabolites (*p* < 0.05) are shown in blue (decreased) and red (increased). *N* = 19 volunteers (one noncompliant excluded) for all graphs.

Following the fasting period, participants underwent 5.3 ± 2.1 days of gradual, medically supervised refeeding with a plant‐based diet (Methods). PCA analysis of plasma metabolite levels post‐refeeding showed substantial overlap with baseline levels, indicating that the fasting‐induced metabolic adaptations were largely transient and reversed upon refeeding (Figure 3A). This is supported by the lack of separation between baseline and post‐refeeding metabolite levels using hierarchical heatmap clustering (Figure 3B). At the individual metabolite level, 109 metabolites (81%) reverted to baseline concentrations after refeeding, while 25 (19%) remained significantly altered (FDR < 5%) with modest effect sizes (|log_2_FC| < 1). Only three metabolites, mannitol, pipecolinic acid, and hippuric acid, were markedly elevated (log_2_FC > 1; Figure 3C). The rise in hippuric acid, a glycine conjugate produced by gut microbial metabolism of dietary polyphenols and a marker of polyphenol intake, likely reflects refeeding with a whole‐food, plant‐based diet. Elevated mannitol levels may similarly signal enhanced gut microbial activity.

Interestingly, although the study was underpowered to detect sex‐specific effects, refeeding revealed significant differences between men and women in two fatty acids. Women exhibited higher post‐refeeding levels of palmitoleic acid and linoleic acid (Figure S1B). Linoleic acid, a polyunsaturated fatty acid (PUFA), has been associated with lower mortality in multiple studies (Harris et al. 2024; Naghshi et al. 2021; Farvid et al. 2014), suggesting a potential beneficial metabolic adaptation in women following fasting.

**FIGURE 4:**
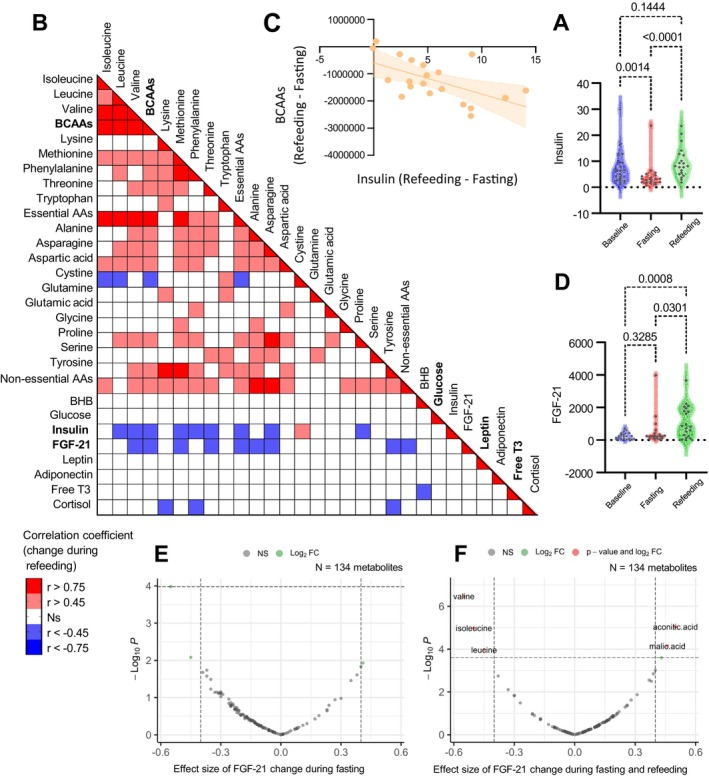
Refeeding stimulates FGF21 production, inversely correlated with BCAAs and methionine. (A) Circulating levels of insulin at three timepoints. Statistical significance was calculated using paired two‐way ANOVA and Turkey's correction for multiple comparisons. *p* Values are shown. Two datapoints were outliers for insulin; graphs without the outliers are shown in Figure S6. (B) Correlation matrix showing Pearson's correlation coefficients (red square = significant positive correlation, blue square = significant negative correlation, *p* value < 0.05) between multiple delta variables (End of Refeeding [ER] – End of Fasting [EF]). Matrix was corrected for outliers. (C) Linear regression between delta insulin and delta BCAAs. Individual dots represent participants. Shaded areas represent 95% CI for the best‐fit line. (D) Circulating levels of FGF21 at three timepoints. Statistical significance was calculated using paired two‐way ANOVA and Turkey's correction for multiple comparisons. *p* Values are shown. Three datapoints were outliers for FGF21; graphs without the outliers are shown in Figure S6. (E, F) Association of FGF21 levels with 134 metabolites by mixed‐effect regression models for longitudinal data, with individuals as the random effect during fasting (E) and refeeding (F). FDR < 0.05.

During refeeding, levels of free T3, glucose, insulin, and leptin increased relative to fasting, likely reflecting a metabolic shift back to glycolysis (Figure 2, Figure 4A). While most amino acids returned to baseline levels (Figure 2A), BCAAs (isoleucine, leucine, and valine) unexpectedly declined, dropping significantly below baseline levels (Figure 2A,B). This reduction correlated significantly with rising insulin levels (*r* = −0.5700, *p* = 0.0087), suggesting insulin‐mediated BCAA uptake into tissues, although correlation does not imply causation (Figure 4B,C). Specifically, changes in insulin levels were strongly correlated with changes in methionine (Pearson *r* = −0.63), leucine (*r* = −0.59), phenylalanine (*r* = −0.55), and valine (*r* = −0.51) (all *p* < 0.05), among others (Figure 4B and Figure S4). Significant correlations are shown in Figure S4. These findings align with previous research showing insulin‐stimulated amino acid transport in skeletal muscle (Saltiel and Kahn 2001; Huang and Czech 2007) and suppression of leucine release during localized hyperinsulinemia (Fryburg et al. 1990), providing a new insight into refeeding amino acid dynamics in humans.

### 2.4 Refeeding Stimulates FGF21 Production, Inversely Correlated With BCAAs and Methionine

Fibroblast growth factor 21 (FGF21) is a hepatokine that plays a critical role in regulating lipid, glucose, and energy metabolism in response to metabolic stress (Luo et al. 2017; Kharitonenkov et al. 2005). It is a well‐known regulator of both prolonged starvation and refeeding (Seo and Kim 2012; Dostálová et al. 2009; Lewis et al. 2019). While fasting rapidly induces FGF21 in mice to modulate the starvation response, humans primarily undergo ketogenesis with a pronounced rise in BHB before FGF21 levels rise (Fazeli et al. 2015). In our study, fasting did not significantly alter plasma FGF21 levels (243.2 to 512.5 pg/mL, adj *p* = 0.3285), showing no consistent increase or decrease in FGF21 (Figure 4D and Figure S6). Even though the 9.8‐day fast may have been insufficient to elicit FGF21 secretion, levels surged upon refeeding to 1176 pg/mL (adj *p* = 0.0007), suggesting that food reintroduction is the primary stimulus (Figure 4D and Figure S6). Several factors may contribute to this increase: low amino acid availability could stimulate FGF21 secretion, as could rising glucose, potentially signaling enhanced uptake. Supporting the first hypothesis, FGF21 during refeeding was inversely correlated with multiple amino acids (all *p* < 0.05), including methionine (*r* = −0.70), alanine (*r* = −0.61), tyrosine (*r* = −0.59), valine (*r* = −0.53), total BCAAs (*r* = −0.46), and others (Figure 4B and Figure S5A). A mixed‐effects regression model for longitudinal data incorporating both fasting and refeeding phases confirmed a significant association between changes in BCAA levels and FGF21 during refeeding, but not during fasting (Figure 4E,F), pointing to refeeding as the mechanistic trigger. These findings align with previous studies showing FGF21 secretion as part of the liver's stress response to BCAA insufficiency (Maida et al. 2017). Additionally, exogenous FGF21 administration has been shown to reduce BCAA accumulation in mice following myocardial infarction (Xu et al. 2022). Inverse associations between methionine and FGF21 have also been documented in both murine (Stone et al. 2014) and human studies (Xiao and Guo 2022; Fangmann et al. 2021). Notably, although FGF21 is known to be elevated in response to hypercaloric carbohydrate‐rich diets (Qian et al. 2022) and has been implicated in glucose uptake regulation in mice (Lewis et al. 2019), we observed no significant correlation between the changes in FGF21 and glucose levels during refeeding (Figure S5B). Instead, our data suggest an association between post‐fast refeeding and FGF21 production, characterized by food‐stimulated insulin secretion, insulin‐associated reductions in circulating BCAA levels through tissue uptake, and concurrent increases in hepatic FGF21.

## 3 Discussion

This study examined the reversible effects of a ~10‐day medically supervised water‐only fast on the human plasma metabolome and endocrine regulators, revealing significant metabolic adaptations across multiple pathways, including ketogenesis, fatty acid oxidation, and amino acid metabolism. These metabolic shifts underscore the physiological mechanisms supporting survival during prolonged fasting and subsequent refeeding. Our findings align with previous evidence demonstrating that diet is a primary determinant of metabolic variability. For example, a large‐scale metabolomic analysis of 1183 plasma metabolites from 1368 individuals in the Netherlands identified dietary intake as a stronger contributor to interindividual metabolic variability than genetic factors (Chen et al. 2022). Similarly, another study analyzing 1251 serum metabolites in 491 healthy individuals confirmed that diet accounted for nearly 50% of the observed metabolic variation (Bar et al. 2020). Consistent with our findings, dietary interventions, including long‐term fasting (Wu et al. 2024) and ketogenic diets (Schweickart et al. 2024), have been shown to profoundly alter the plasma metabolome. This is particularly important, as the plasma metabolome serves as a dynamic biomarker of an individual's health status, with the potential to predict disease progression and mortality, and biological age (Zhang et al. 2024; Buergel et al. 2022; Talmor‐Barkan et al. 2022).

Among the metabolites altered by prolonged fasting in our study, seven (i.e., glucose, 3‐hydroxybutyric acid, glycine, glutamine, alanine, phenylalanine, and tyrosine) have been previously identified as aging‐associated biomarkers in one of the most comprehensive metabolomic studies on all‐cause mortality, which analyzed 325 circulating biomarkers from 250,341 UK Biobank participants (Zhang et al. 2024). Our findings suggest that fasting and refeeding cycles may modulate the aging metabolome; however, the short‐term nature of our study limits interpretation of long‐term effects. Despite the small sample size, our results present evidence of potential sex‐specific variations in linoleic acid levels that warrant further investigation. Linoleic acid has been associated with improved metabolic health and reduced mortality risk (Harris et al. 2024). In the UK Biobank study, the linoleic acid‐to‐total fatty acids ratio corresponded to the lowest hazard ratio for all‐cause mortality, suggesting that post‐fast refeeding may confer metabolic benefits, particularly in women (Zhang et al. 2024).

BCAAs have garnered significant interest due to their complex relationship with metabolic health and longevity (Choi et al. 2024; Green and Lamming 2019; Green et al. 2022; Cummings et al. 2018). In our study, fasting and refeeding exerted distinct effects on BCAAs, with levels remaining stable during fasting but unexpectedly declining during refeeding. While the maintenance of BCAA levels during starvation has been well documented (Steinhauser et al. 2018), the marked reductions in BCAAs upon refeeding represent a novel finding. Stable circulating BCAAs during fasting may reflect reduced oxidation or continued release from peripheral tissues. Previous starvation studies in both rats and humans have implicated the liver (Holecek et al. 2001) and skeletal muscle (Fryburg et al. 1990) as sources of circulating BCAA. Future work should delineate how muscle mass and nitrogen balance contribute to plasma BCAA dynamics. Preclinical studies suggest that dietary BCAA restriction may enhance metabolic health by increasing energy expenditure and reducing adiposity (Solon‐Biet et al. 2022; Fontana et al. 2016). Specifically, isoleucine restriction has been linked to lifespan extension (Green et al. 2023), while concurrent restriction of isoleucine and valine has further improved metabolic health (Yu et al. 2021). In human cohorts, a systematic review and meta‐analysis revealed that elevated plasma levels of certain amino acids, including BCAAs, are linked to an increased risk of type 2 diabetes. (Morze et al. 2022) Interventional clinical trials have corroborated these findings, demonstrating that dietary BCAA restriction reduces plasma BCAA levels, decreases fasting insulin concentration, and improves insulin sensitivity in both healthy volunteers (Ramzan et al. 2020), and those with type 2 diabetes (Karusheva et al. 2019). These findings align with previous research linking high BCAA levels to an elevated risk of metabolic dysfunction (Buergel et al. 2022), suggesting that lower BCAA levels may be metabolically advantageous.

While elevated BCAA levels have been linked to metabolic dysfunction, certain studies also suggest a potential protective role in aging and neurodegenerative diseases. The UK Biobank study identified 25 potentially “anti‐aging” metabolites, including four amino acids (valine, histidine, glycine, and leucine), two of which are BCAAs (Zhang et al. 2024), suggesting a potential protective role for BCAAs in aging. Additionally, a large prospective study involving 22,623 participants found that lower BCAA levels were associated with an increased risk of both overall dementia and Alzheimer's disease (Tynkkynen et al. 2018). These findings suggest that BCAA metabolism may have complex, context‐dependent effects on health, varying with age and disease state (Choi et al. 2024; Yao et al. 2023). However, the precise role of BCAAs in human longevity and disease risk remains unresolved, necessitating further investigation.

Our findings highlight the refeeding phase following fasting as a critical window for modulating BCAA levels. We observed a significant reduction in BCAA levels during refeeding, which strongly correlated with increased FGF21 production, a key hormone that enhances glucose uptake and promotes energy expenditure (Luo et al. 2017; Kharitonenkov et al. 2005). FGF21 has been proposed to respond to several nutritional cues, including low insulin and increased ketogenesis, as well as low dietary protein intake. In rodent models, fasting rapidly induces hepatic FGF21 to orchestrate the starvation response, whereas in humans, ketogenesis typically precedes any rise in FGF21 (Fazeli et al. 2015). Our data support the hypothesis that FGF21 secretion is primarily triggered by BCAA insufficiency. During fasting, circulating BCAA concentrations remained stable, and FGF21 levels did not change, despite reductions in glucose and insulin and an increase in ketogenesis. In contrast, refeeding led to a decline in BCAA levels accompanied by a parallel increase in FGF21, even as glucose, insulin, and ketones returned toward baseline. These observations suggest that reduced BCAA availability serves as a key signal for FGF21 induction, which may in turn confer metabolic benefits through improved glycaemic regulation. Notably, recent clinical trials on FGF21 analogs have demonstrated promising metabolic benefits, including reductions in fasting insulinemia, body weight, and total cholesterol (Yan et al. 2021; Carbonetti et al. 2023; Zhang and Li 2015; Crunkhorn 2013). Additionally, protein intake‐induced insulin resistance has been associated with altered FGF21 metabolism, suggesting a complex regulatory interplay between amino acid intake and FGF21 signaling (Harris et al. 2017). Importantly, the adverse metabolic effects of BCAAs appear to be primarily mediated by isoleucine and valine (Yu et al. 2021), reinforcing the potential metabolic benefits of reducing these amino acids during refeeding. Therefore, the metabolic stress imposed by fasting and refeeding may activate protective pathways, such as FGF21 secretion, which could be particularly beneficial for individuals with metabolic disorders, including obesity and metabolic syndrome. Given the ongoing debate regarding BCAAs' role in aging and disease, future research should further elucidate the long‐term health implications of fasting‐induced BCAA modulation, considering factors such as age, metabolic status, and disease susceptibility.

### 3.1 Strengths and Limitations

This study has several strengths, including in‐clinic monitoring of adherence via BHB measurement to ensure compliance, 24/7 medical supervision, a longitudinal study design that accounts for differences in baseline values, and the integration of metabolomics with multiple endocrine regulators. However, there are limitations. The relatively small sample size limits the ability to detect sex‐ and age‐specific effects and precludes detailed comparisons across metabolic subgroups. Additionally, variability in fasting and refeeding durations, as participants chose their preferred lengths, and the lack of randomization may introduce confounding. Future studies should specifically examine how baseline metabolic status (such as hyperglycaemic, hyperinsulinaemic, and obesity) modulates responses to fasting and refeeding.

## 4 Conclusion

In summary, prolonged fasting induces profound yet reversible metabolic shifts, with significant alterations in amino acid metabolism, fatty acid oxidation, and endocrine signaling. The refeeding phase presents a critical metabolic transition, characterized by a reduction in circulating BCAA levels and increased FGF21 production. These findings provide novel insights into the physiological adaptations to fasting and suggest that the metabolic benefits of fasting may be partially mediated through post‐fast refeeding dynamics. Future research should explore the long‐term implications of fasting‐induced BCAA reductions and their potential interactions with FGF21 signaling in metabolic health and aging.

## 5 Methods

### 5.1 Study Participants

The study protocol was approved by the institutional review board at Marin General Hospital in Greenbrae, California, USA, and conducted in accordance with the Declaration of Helsinki. The study was conducted at the TrueNorth Health Center, a private facility that provides medically supervised fasting. The study team, which worked independently from the Center, invited potential volunteers to participate in the study, and those who expressed interest were screened for eligibility. All participants provided written informed consent prior to enrollment. Of the 168 individuals screened, 33 met the inclusion criteria (age ≥ 18 years). Exclusion criteria included a history of chronic disease, physical or psychiatric disorders, medication use interfering with fasting, and lifestyle factors affecting adherence, such as alcoholism. Of the eligible individuals, 20 participants (*n* = 20) consented to and completed a medically supervised water‐only fasting protocol. Blood samples were collected after an overnight fast, and analyzed for metabolomic profiling, a secondary outcome of the study. The primary outcome, systemic inflammation assessed via C‐reactive protein levels, has been previously reported with intervention details (Commissati et al. 2025).

### 5.2 Medically Supervised, Water‐Only Fasting, and Refeeding Protocol

The water‐only fasting and refeeding protocol was conducted at TrueNorth Health Center in Santa Rosa, California, a facility specializing in prolonged water‐only fasting. The program was medically supervised, with physicians performing comprehensive physical, neurological, and psychological assessments. Data collection and measurements were independently conducted by Dr. Serena Commissati between May and December 2017. Prior to fasting, participants underwent a thorough medical evaluation, including a review of medical history, a comprehensive metabolic panel and blood count, and urinalysis. In the 2 days preceding the fast, participants were restricted to a diet of fresh raw fruits and steamed vegetables. During the fasting period, participants remained at the facility, consuming a minimum of 1182 mL of water daily while minimizing physical activity. Vital signs and symptoms were monitored twice daily by medical staff, with urinalysis and blood tests performed weekly or as clinically indicated. Fasting discontinued based on symptom stabilization, participant request, or medical necessity. Refeeding was introduced gradually, beginning with juice on the first day, followed by the progressive introduction of whole‐plant foods free from added sugars, oils, and salts. Moderate physical activity was reintroduced incrementally, with clinicians providing twice‐daily monitoring throughout the refeeding phase.

### 5.3 Blood Analyses

Venous blood samples were collected from all participants following an overnight fast at multiple time points: baseline (BL), week 1 fasting (week 1), end of fasting (EF), and end of refeeding (ER). For participants who fasted for 1 week, EF = week 1. In cases where volunteers consumed vegetable juice or broth in the final days of fasting, their week 1 samples were included in the analysis. Blood was collected in non‐gel serum tubes and EDTA tubes, centrifuged at 4°C, and serum and plasma were aliquoted and stored at −80°C until analysis. To minimize inter‐assay variation, all samples from the same participant were analyzed within the same batch. Serum and plasma analyses were conducted at the Core Laboratory for Clinical Studies at Washington University in St. Louis. Technicians, blinded to the time point assignments, performed all assessments. Hormonal measurements (including insulin, FGF21, leptin, adiponectin, free T3, and cortisol) were conducted using commercially available Enzyme‐Linked Immunosorbent Assay (ELISA) Quantikine kits from R&D Systems Inc., Minneapolis, MN, with intra‐ and inter‐assay coefficients of variation < 10%.

### 5.4 Metabolomics Acquisition

Sixty plasma samples collected from 20 participants at three timepoints (Baseline, Fasting, and Refeeding) were included in the metabolomic analysis. Metabolite identification was performed using ALEX‐CIS GCTOF MS (Automated Liner Exchange‐Cold Injection System‐Gas Chromatography Time Of Flight Mass Spectrometer), with analysis conducted by technicians at the NIH West Coast Metabolomics Center. Data acquisition followed previously established protocols (Fiehn et al. 2008). The analytical GC column (Restek Corporation Rtx‐5Sil MS) was operated at 50°C–330°C, mobile phase helium, flow rate of 1 mL/min. The injection volume (0.5 μL) was injected 25 splitless times into a multi‐baffled glass liner at 50°C and ramped to 250°C by 12°C/s. The oven temperature was 50°C for 1 min, then ramped at 20°C/min to 330°C, and held constant for 5 min. The chromatography method was optimized for retention and separation of primary metabolite classes (amino acids, hydroxyl acids, carbohydrates, sugar acids, sterols, aromatics, nucleosides, amines, and miscellaneous compounds). The mass spectrometer (Leco Pegasus IV) was operated with unit mass resolution at 17 spectra/s from 80 to 500 Da at −70 eV ionization energy and 1800 V detector voltage with a 230°C transfer line and a 250°C ion source. Raw data files were processed in ChromaTOF version 2.32. The processing involved no smoothing, a 3‐s peak width, baseline subtraction just above the noise level, and automatic mass spectral deconvolution and peak detection at signal/noise levels 5:1 throughout the chromatogram. Raw data files are securely stored at the NIH Metabolomics database (www.metabolomicsworkbench.org).

### 5.5 Metabolomics Analysis

The normalized peak height (Fiehn Lab) values of the 134 identified metabolites (285 metabolites were unknowns of a total of 419 metabolites measured) were analyzed using MetaboAnalyst 6.0 (www.metaboanalyst.ca). For all analyses, the peak height values for *n* = 20 participants were grouped by baseline (BL), end of fasting (EF), and end of refeeding (ER) study stages, or by gender. Data were log‐transformed (base 10) prior to downstream analysis. To identify potential outliers or distinct clusters among participants, principal component analysis (PCA) was employed, with results presented with 95% confidence intervals around the mean, displayed as ellipses. Then, hierarchical clustering was conducted using Ward's minimum variance method (Murtagh and Legendre 2014) with Pearson's distance measure, and the results were visualized using a heatmap. A pairwise metabolome‐wide comparison across the three categories was performed using paired Student's *t*‐tests. Significance was set at *p* < 0.05, with a log2 fold change (log2FC) greater than 1 or less than −1, indicating either a doubling or halving of metabolite levels. Results were visualized using volcano plots.

### 5.6 KEGG Pathway Enrichment Analysis

To identify KEGG pathways significantly over‐represented, KEGG pathway enrichment analysis was applied among the metabolites showing a significant increase or decrease, as determined by the metabolome‐wide analysis. The KEGG human metabolic pathways database (December 2023) was used as the metabolite library, and only pathways with at least two entries were included in the enrichment analysis.

### 5.7 Ingenuity Pathway Analysis

Alterations in canonical pathways were generated with IPA software (QIAGEN Inc., https://www.qiagenbioinformatics.com/products/ingenuity‐pathway‐analysis) using the metabolomics dataset as an input (*n* = 134 metabolites), including metabolite names, fold‐changes (fasting/baseline), and adjusted *p* values. IPA mapped 114 entities (114/134 = 85%) that were analyzed using Metabolomics Analysis based on log2(FC) values. The adjusted *p* value cutoff was 0.01 and the log2(FC) cutoffs were −1 to 1, producing 18 analysis‐ready molecules (15 downregulated and 3 upregulated).

### 5.8 CompBio Analysis

Metabolites differentially expressed across dietary conditions from the statistical analysis were analyzed using the CompBio platform (https://gtac‐compbio‐ex.wustl.edu). CompBio is an AI platform to analyze multi‐omics data by identifying enriched biological themes from literature. Interactive 3D knowledge maps were generated where significant themes (Normalized Enrichment Score > 1.2 and *p* value < 0.1) were represented as spheres, sized and ranked based on overall absolute enrichment between associated entities.

### 5.9 Statistical Analyses

Changes in absolute variables (free T3, glucose, leptin, insulin, FGF21, BHB, adiponectin, and cortisol) and the 18 amino acids detected through metabolomics were calculated across three timepoints: baseline (BL), fasting (EF), and refeeding (ER) for all participants. Statistical significance for each variable was determined using paired two‐way ANOVA, with Geisser–Greenhouse correction for sphericity and Tukey's correction for multiple comparisons. Significance levels were indicated by asterisks: *p < 0.05, **p < 0.01, ***p < 0.001, and ****p < 0.0001. Correlation analyses were performed using Pearson's correlation for normally distributed variables. Two correlation matrices were generated to assess the relationships between amino acid level changes (“Fasting – Baseline” and “Refeeding – Fasting”) and the changes in other measured variables during the corresponding intervals. Statistical significance for correlations was labeled at *p* < 0.05 and p < 0.01, using GraphPad Prism 10. Delta values between timepoints were used instead of individual measurements to capture the dynamics of variable changes. For FGF21, its correlation with the circulating metabolome was assessed using mixed‐effects regression models for longitudinal data using R software (version 4.2.1) and RStudio, with individuals treated as a random effect. A False Discovery Rate (FDR) correction was applied to adjust for multiple testing and control the type I error rate at 5%. Spelling and grammar were checked using LLMs; however, all writing is original.

## Author Contributions

L.F. and B.K.K.: Study design, supervision, and funding acquisition. A.G.: Study facilities. S.C.: Recruitment, monitoring, and sample collection. M.L.C., N.T.S., G.F., and A.M.: Data analysis and figures. M.L.C.: Manuscript draft. A.M., A.J.H., N.T.S., G.R., G.F., L.F.: Manuscript revision.

## Conflicts of Interest

A.G. is the founder of TrueNorth Health Center, a private facility that offers medically supervised fasting interventions. The other authors report no conflicts of interest. The funding sources were not involved in any form in the findings presented in the study. The article was not commissioned. No author was precluded access to data.

## Supporting information


**Figure S1:** (A) PCA for metabolite levels in women (pink) and men (blue) at BL, EF, and ER. Overlap in colored areas suggests similarity in metabolite levels between genders. (B) Volcano plots comparing metabolite levels between women (variable) and men (reference) at EF and ER. Significantly changed metabolites (*p* < 0.05) are shown in blue (decreased) and red (increased).
**Figure S2:** (A) Compbio theme analysis of metabolites increased or decreased following fasting. Analyses were performed with *p* < 0.05 between EF and BL and *n* = 19 participants. (B) Ingenuity Pathway Analysis (IPA) of canonical pathways significantly increased (red) or decreased (green). (C) IPA predicted suppression of protein and amino acid synthesis (blue) and predicted increase of amino acid uptake (orange) during fasting based on significantly expressed metabolite levels.
**Figure S3:** Variables significantly correlated with changes in free T3 levels during fasting. Participants are represented by individual dots. Shaded areas represent 95% CI for the best fit line.
**Figure S4:** Variables significantly correlated with changes in insulin levels during refeeding. Participants are represented by individual dots. Shaded areas represent 95% CI for the best fit line.
**Figure S5:** (A) Variables significantly correlated with changes in FGF21 levels during refeeding. Participants are represented by individual dots. Shaded areas represent 95% CI for the best fit line. (B) Lack of correlation between the increase in FGF21 and the increases in glucose and insulin during refeeding. Participants are represented by individual dots (glucose) and squares (insulin).
**Figure S6:** Comparison graphs with and without statistical outliers.

**Data S1:** acel70270‐sup‐0002‐DataS1.xlsx

## Data Availability

Metabolomics data is available as an Excel file Data S1.
