# Supplementary material for: The Metabolic Transition Between Fasting and Feeding Alters Aging‐Associated Metabolites, Lowers BCAAs, and Stimulates FGF21 Production in Humans
Source: Aging Cell. 2025 Oct 21;24(12):e70270. doi: 10.1111/acel.70270 (PMC12686557; doi:10.1111/acel.70270)
Supplement: Supplementary file 1 — Figure S1: (A) PCA for metabolite levels in women (pink) and men (blue) at BL, EF, and ER. Overlap in colored areas suggests similarity in metabolite levels between genders. (B) Volcano plots comparing metabolite levels between women (variable) and men (reference) at EF and ER. Significantly changed metabolites (p < 0.05) are shown in blue (decreased) and red (increased). Figure S2: (A) Compbio theme analysis of metabolites increased or decreased following fasting. Analyses were performed with p < 0.05 between EF and BL and n = 19 participants. (B) Ingenuity Pathway Analysis (IPA) of canonical pathways significantly increased (red) or decreased (green). (C) IPA predicted suppression of protein and amino acid synthesis (blue) and predicted increase of amino acid uptake (orange) during fasting based on significantly expressed metabolite levels. Figure S3: Variables significantly correlated with changes in free T3 levels during fasting. Participants are represented by individual dots. Shaded areas represent 95% CI for the best fit line. Figure S4: Variables significantly correlated with changes in insulin levels during refeeding. Participants are represented by individual dots. Shaded areas represent 95% CI for the best fit line. Figure S5: (A) Variables significantly correlated with changes in FGF21 levels during refeeding. Participants are represented by individual dots. Shaded areas represent 95% CI for the best fit line. (B) Lack of correlation between the increase in FGF21 and the increases in glucose and insulin during refeeding. Participants are represented by individual dots (glucose) and squares (insulin). Figure S6: Comparison graphs with and without statistical outliers. [file ACEL-24-e70270-s001.pdf]

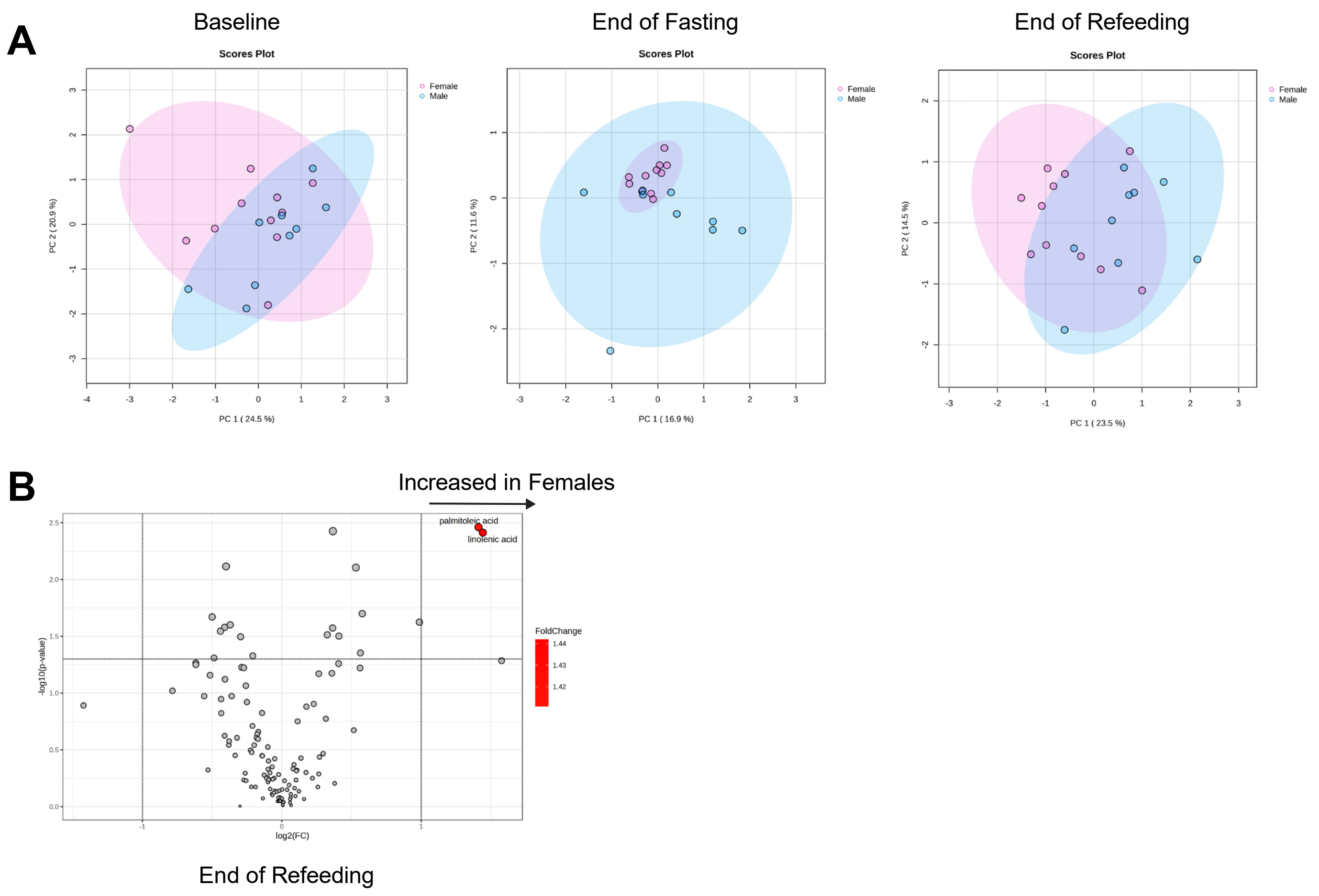

Supplementary Figure 1

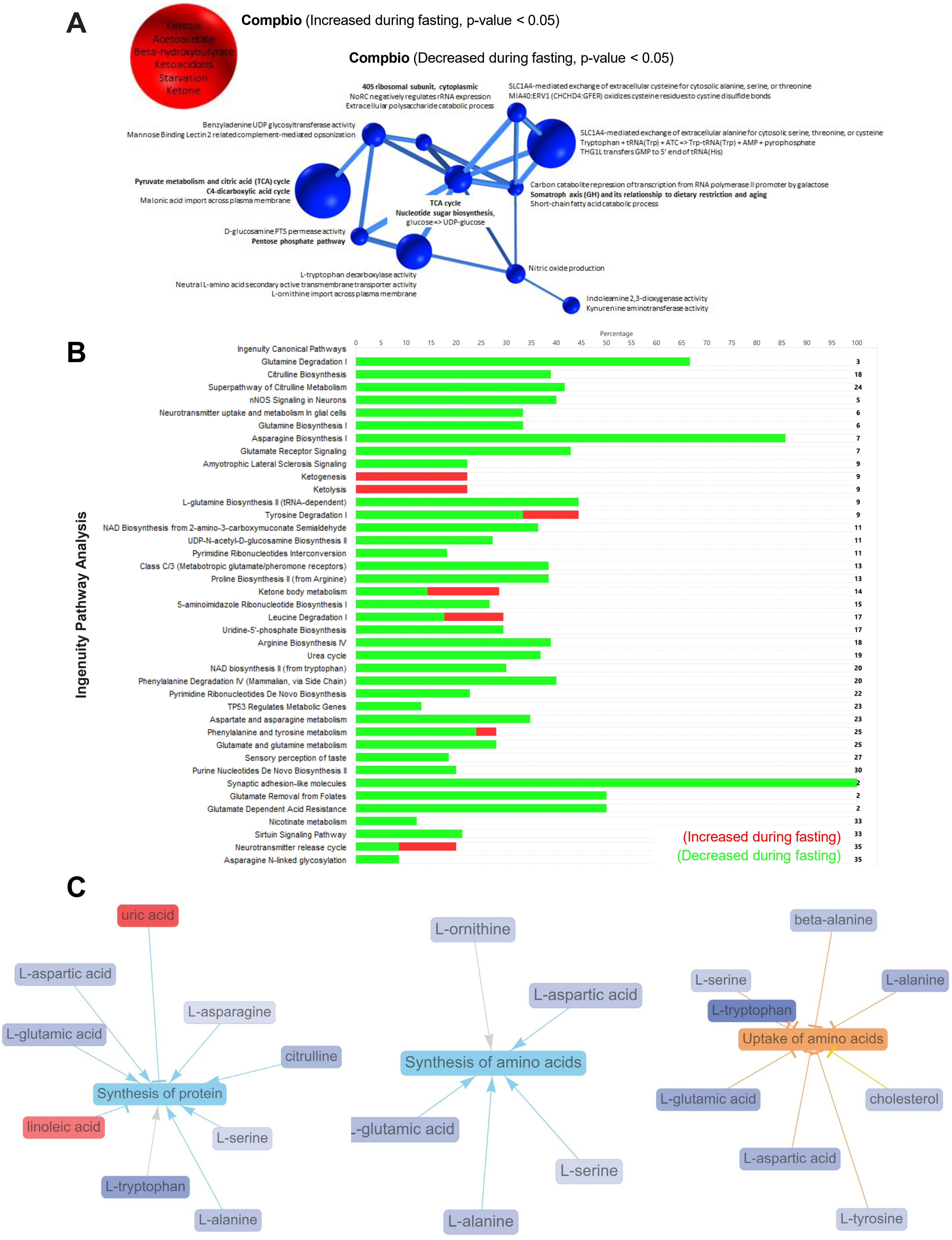

Supplementary Figure 2

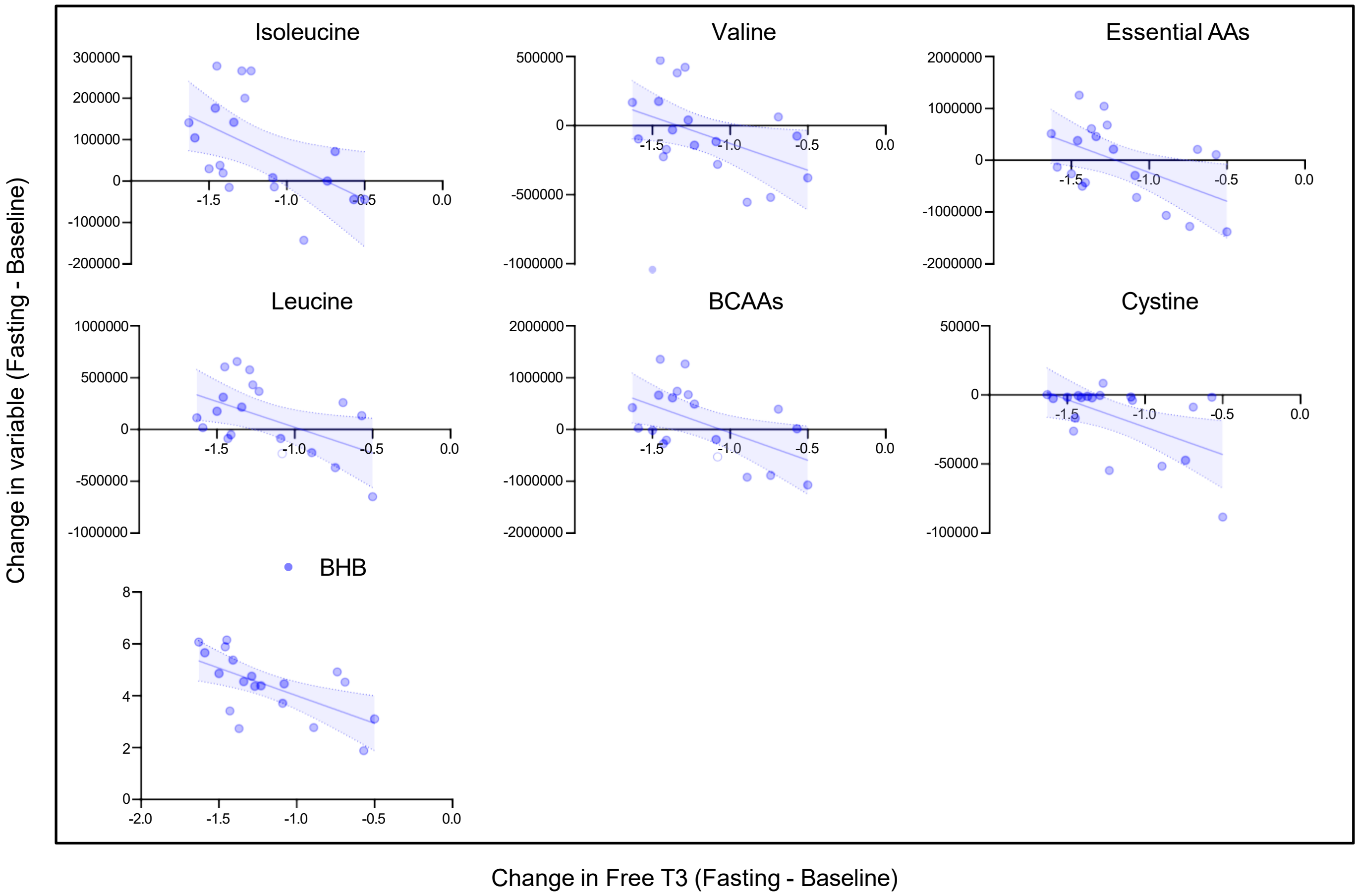

Supplementary Figure 3

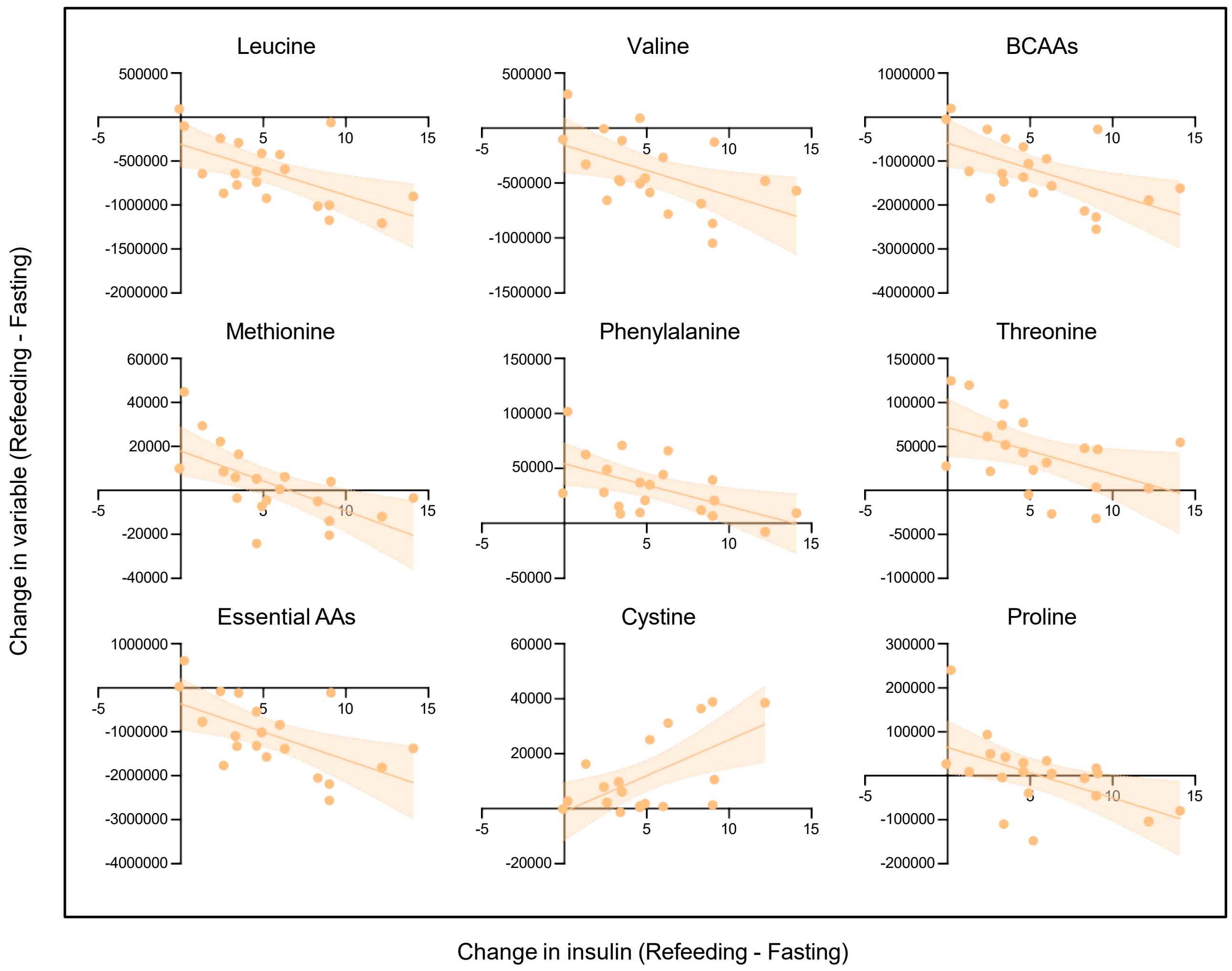

Supplementary Figure 4

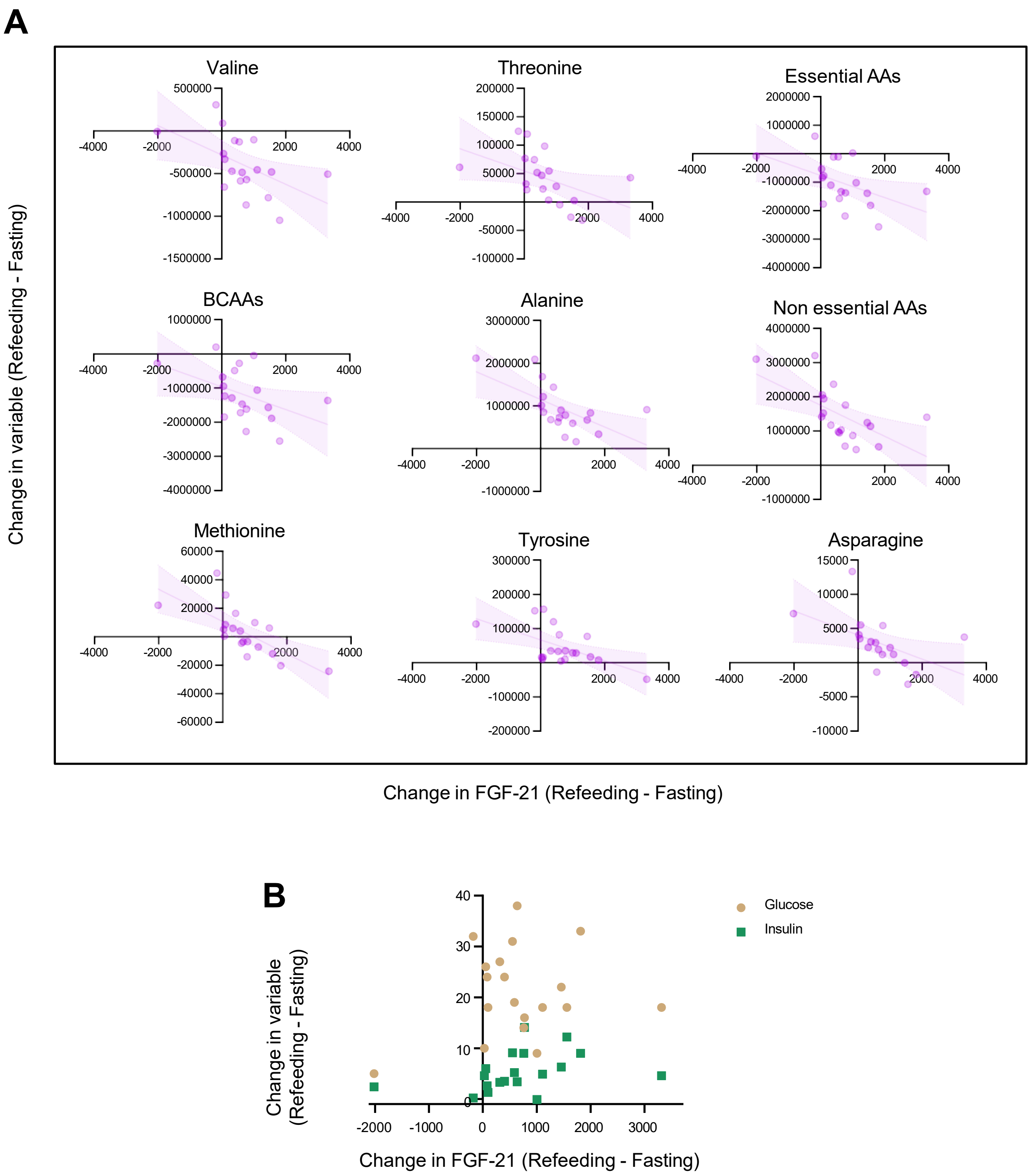

Comparator: Refeeding FGF-21	Glucose	Insulin
Pearson's R squared	0.03466	0.123
P value	0.4454	0.141
Deviation from zero?	Not Significant	Not Significant

Supplementary Figure 5

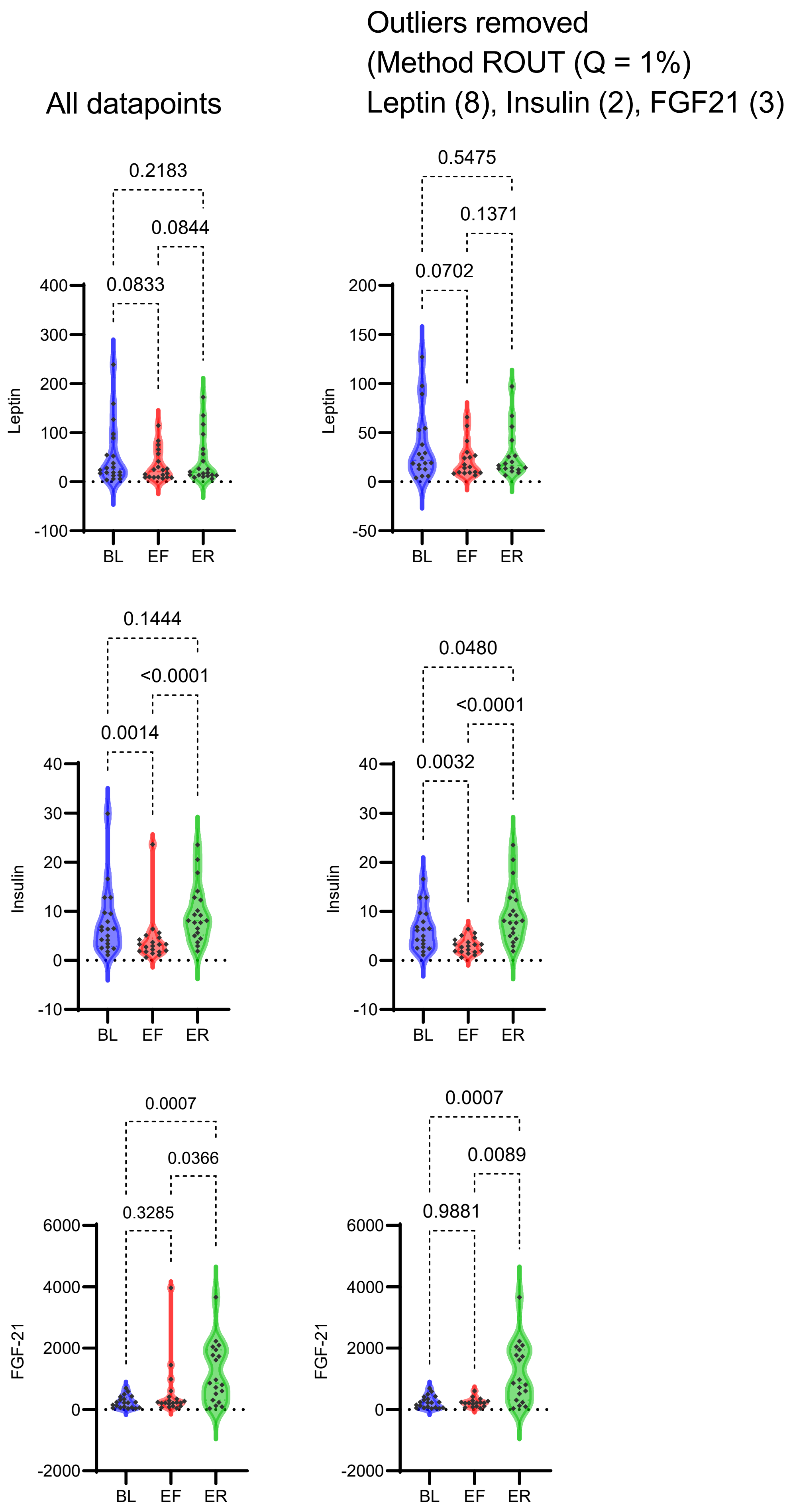

Supplementary Figure 6
